# Thyroid hyalinizing trabecular adenoma with a high thyroglobulin level: a case report

**DOI:** 10.1093/jscr/rjab324

**Published:** 2021-07-31

**Authors:** Hirofumi Katano, Hisashi Hasegawa, Hiroumi Matsuzaki, Takeshi Oshima, Xiaoyan Tang

**Affiliations:** Department of Otolaryngology-Head and Neck Surgery, Nihon University Hospital, 1-6 Kandasurugadai, Chiyoda-ku, Tokyo 101-8309, Japan; Department of Otolaryngology-Head and Neck Surgery, Nihon University Hospital, 1-6 Kandasurugadai, Chiyoda-ku, Tokyo 101-8309, Japan; Department of Otolaryngology-Head and Neck Surgery, Nihon University Hospital, 1-6 Kandasurugadai, Chiyoda-ku, Tokyo 101-8309, Japan; Department of Otolaryngology-Head and Neck Surgery, Nihon University Hospital, 1-6 Kandasurugadai, Chiyoda-ku, Tokyo 101-8309, Japan; Department of Pathology, Nihon University School of Medicine, 1-6 Kandasurugadai, Chiyoda-ku, Tokyo 101-8309, Japan

## Abstract

Thyroid hyalinizing trabecular adenoma (HTA) is a rare and borderline tumor of follicular origin. It is characterized by a trabecular growth pattern and marked intratrabecular hyalinization. Excessively elevated thyroglobulin levels have not been reported previously in cases without bilateral lung metastases. Here, we present a case of a 54-year-old woman with chronic thyroiditis with a 50-mm tumor in the left lobe of the thyroid gland, which was observed on ultrasonography. Her thyroglobulin level was found to be elevated at 684 ng/ml. Since fine needle aspiration cytology could not exclude possible malignancy, she underwent thyroid lobectomy; the final diagnosis was thyroid HTA. Two weeks after resection, her thyroglobulin level showed negative conversion. To our knowledge, this is the first report of a patient with a thyroid HTA exhibiting a thyroglobulin level as high as that for a patient with hyalinizing trabecular carcinoma.

## INTRODUCTION

Thyroid hyalinizing trabecular adenoma (HTA), which was first reported by Carney *et al*. in 1987 [1], is a rare tumor. According to the World Health Organization, it is classified as a borderline and uncommon tumor of follicular origin, with a trabecular pattern of cell growth and marked intratrabecular hyalinization [2]. This tumor type accounts for <1% of all thyroid tumors [3], has a male-to-female incidence ratio of 1:6 and most commonly presents in individuals in their fifties [3, 4]. This disease is also painless, and is generally found incidentally on ultrasonography (US) if the lesion size is <30 mm [4]. Histologically, tumor cells are characterized by yellow bodies in the cytoplasm (periodic acid-Schiff [PAS]-positive staining) and a glass-like substance with positivity for collagen type IV [5]. Ki-67-positive tumor cells on the cell membrane in papillary thyroid carcinoma are useful in differentiating it from papillary thyroid cancer [6]. Although the clinical course of this lesion is generally benign, lymph node and other organ metastases have occurred in some patients [4, 7, 8].

**Figure 1:**
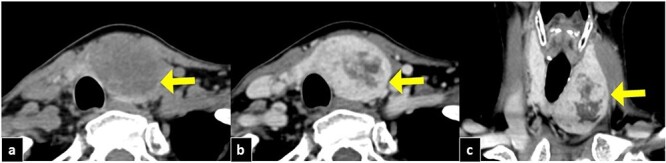
Cervical CT imaging. The tumor is well defined in the left lobe of the thyroid gland, with a size of 50 mm. (**a**) CT section in the axial section, showing a tumor with low density (arrow). (**b**) Axial section with gadolinium contrast (arrow). (**c**) Coronal section with gadolinium contrast (arrow).

**Figure 2:**
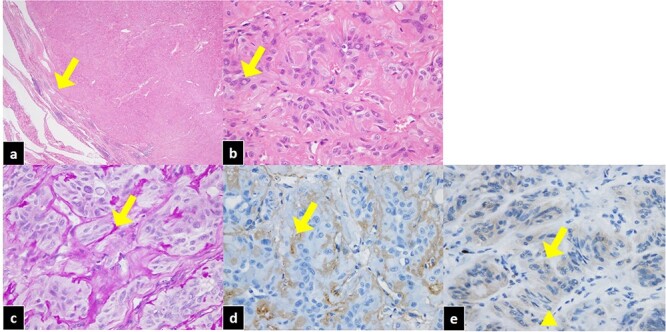
Pathological features of HTA of the thyroid. (**a**) The tumor is well circumscribed with a thin fibrous capsule (arrow). Hematoxylin and eosin staining, ×20. (**b**) Spindle-shaped and intranuclear inclusions (arrow) can be identified. Hematoxylin and eosin staining, ×400. (**c**) PAS-positive hyalinizing material can be seen between the trabecular structures (arrow). PAS staining, ×400. (**d**) Hyalinization is positive (arrow). Collagen type IV staining, ×400. (**e**) Ki-67 staining was positive for cytoplasm and negative for the cell membrane (arrow). The nucleus was nearly negative (triangle).

Clinical management of this disease can be challenging because of the inherent difficulty in obtaining a preoperative diagnosis. These tumors are generally painless and soft without clinical features. In addition, preoperative diagnostic imaging, including US and computed tomography (CT), which generally revealed solid pattern, does not usually demonstrate any characteristic features [9]. In fact, only ~29% of the patients with this disease reportedly demonstrate malignant features on the US [10]. Fine needle aspiration (FNA) cytology, which is highly sensitive, can fail to provide an accurate diagnosis, occasionally resulting in a misdiagnosis of papillary thyroid carcinomas [6, 11] and unnecessary surgery [4]. Excessive elevations in thyroglobulin levels have not previously been reported in cases without metastases. To our knowledge, no report on thyroglobulin levels in patients with HTAs exists.

Here we present a case involving a middle-aged woman with a thyroid HTA with unusual US and Ki-67 pattern whose preoperative thyroglobulin level was as high as that of a patient with a thyroid hyalinizing trabecular carcinoma with bilateral lung metastases.

## CASE REPORT

A 54-year-old woman was admitted to our hospital for the management of a growing, 50-mm, painless, elastic, left cervical mass. The patient had a medical history of panic disorder, with no history of alcohol abuse, smoking, or drug allergies. Blood laboratory examinations demonstrated normal thyroid stimulating hormone (TSH), free triiodothyronine (fT3), and free thyroxine (fT4) levels, elevated thyroglobulin level of 684 ng/mL (normal, ≤33.7 ng/mL), elevated anti-thyroid microsomal antibody level of 334 IU/mL (normal, <16 IU/mL), and elevated anti-thyroglobulin antibody level of 175 IU/mL (normal, <28 IU/mL). The patient was diagnosed with chronic thyroiditis. Chest radiography ruled out tumor growth in either lung.

On US, a heterogeneous mass showing a well-defined border, measuring 41 × 45 × 24 mm (width × length × depth), with a cystic internal lesion was identified in the left lobe of the thyroid gland. On CT (Fig. 1), the mass showed a low density with enhancement similar to that of the thyroid parenchyma, except in the cystic portion. In addition, the trachea was displaced to the right by the tumor. FNA revealed chronic thyroiditis on two occasions, and the possibility of malignancy could not be ruled out. Accordingly, left thyroid lobectomy was performed.

The resected specimen showed an encapsulated tumor measuring 25 × 21 × 35 mm (width × length × depth), with evidence of cystic degeneration. The tumor cells possessed abundant eosinophilic cytoplasm, oval to polymorphic nuclei and many intranuclear inclusion bodies (Fig. 2). PAS-positive puncta with immunohistochemical positivity for collagen type IV were found between the trabecular structures. No yellow bodies were observed. Ki-67 was positive for cytoplasm and negative for the cell membrane, and the nucleus was nearly negative. The histopathological examination revealed an HTA.

Two weeks following the surgery, the thyroglobulin level showed negative conversion and was detected as 14.2 ng/ml on the first postoperative exam. Eighteen months later, at the time of writing this report, there was no evidence of recurrence or metastasis with negative thyroglobulin level.

Informed consent for publication of this report was obtained from the patient.

## DISCUSSION

To our knowledge, this is the first reported patient with such a high thyroglobulin level (684 ng/ml) associated with an adenoma with chronic thyroiditis; moreover, this is the first reported patient with a thyroglobulin level as high as that of a patient with a thyroid hyalinizing trabecular carcinoma and bilateral lung metastases (622 ng/ml) [7]. There have been no case reports of prominent elevation of thyroglobulin to this level in thyroid HTAs. The effect of chronic thyroiditis on the high thyroglobulin level in this case was considered on studying the specific US and pathological features.

We report an unusual case of a thyroid HTA with a high preoperative thyroglobulin level that was comparable to that of a patient with metastatic carcinoma. Moreover, there were several specific features in this case. First, this case showed a cystic pattern on US, while most HTAs show a solid pattern [6]. Anti-thyroid microsomal antibody and anti-thyroglobulin antibody could have influenced this cystic pattern on US and excessive high thyroglobulin level in this case. Second, pathological features in this case were unusual. In this case, Ki-67 immunohistochemical staining was positive for cytoplasm and negative for the cell membrane. The nucleus was also negative. Polymorphic nuclei are unusual in HTAs [6]. Ki-67 staining of thyroid HTAs is characterized by being strongly positive on the cell membrane and nucleus [6]. Moreover, the pathological features similar to the ones in this case have been observed in papillary thyroid carcinoma [6]. Therefore, the pathology of thyroid HTAs with high thyroglobulin levels and chronic thyroiditis should be carefully evaluated for precise diagnosis and appropriate management.

## AUTHORS’ CONTRIBUTIONS

H.K.: manuscript writing; H.H.: project development, data collection, manuscript writing; H.M.: manuscript editing; T.O.: manuscript editing; X.T.: manuscript editing.
